# Transferrin Promotes Oligodendroglial Differentiation Independently of Iron Binding and Release

**DOI:** 10.1080/17590914.2026.2720203

**Published:** 2026-08-24

**Authors:** Estefania Chamorro-Aguirre, Martina Garmendia, María Julia Pérez, Paula G. Franco, Agustín Byrne, Hugo Adamo, Jorge Correale, Juana María Pasquini

**Affiliations:** a Facultad de Farmacia y Bioquímica. Departamento de Química Biológica, Universidad de Buenos Aires, Buenos Aires, Argentina; b CONICET. Instituto de Química y Fisicoquímica Biológicas (IQUIFIB), Buenos Aires, Argentina; c Departamento de Neurología FLENI, Buenos Aires, Argentina

**Keywords:** Iron, maturation, oligodendrocyte, transferrin

## Abstract

Transferrin (Tf), classically known as the major iron-transport protein in mammals, also engages in potent promyelinating activity within the central nervous system by accelerating oligodendrocyte maturation. Since 1994, our group has shown that intrathecal or intranasal administration of apoTransferrin (aTf) promotes oligodendroglial differentiation and myelin protein expression *in vivo* and *in vitro*. A long-standing question, however, has been whether these effects depend on iron delivery or instead reflect an intrinsic trophic property of the protein. To directly address this point, we used the oligodendroglial precursor cell line Oli-Neu and two previously characterized non-glycosylated human Tf mutants with selective defects in iron handling: TfY (Y95F/Y188F/Y426F/Y517F), an iron-binding-deficient mutant unable to bind iron, in either lobe, and TfK (K206E/E534A), an iron-release-deficient mutant that binds iron but cannot release it. We first confirmed that Oli-Neu cells express endogenous Tf and Tf receptor 1 (TfR1), internalize exogenous human Tf, and display low, non-colocalizing TfR2 expression. Transient expression of wild-type Tf, TfY, or TfK increased myelin basic protein (MBP) immunoreactivity and induced complex oligodendrocyte-like morphologies compared with non-transfected cells. Both mutants promoted maturation despite their opposite iron-binding defects, with TfK generating highly branched arbors and TfY inducing a more compact process architecture. AlphaFold-based structural modelling demonstrated the preservation of the canonical bilobalTf scaffold in both mutants. These findings provide direct evidence that the promyelinating and promaturational actions of Tf do not require iron binding or release, but rather reside in the protein itself. By decoupling Tf’s biological activity from iron metabolism, this work expands the current understanding of Tf biology and identifies Tf as an iron-independent signaling cue for oligodendrocyte differentiation.This insight opens new avenues for developing Tf-based or Tf-mimetic therapeutic strategies to enhance remyelination in demyelinating disorders such as multiple sclerosis.

## Introduction

Transferrin (Tf) is classically recognized as the principal iron-transport protein in mammals, with highest expression in the liver. However, Tf is also synthesized within the central nervous system (CNS), particularly by oligodendrocytes (OLGs) and choroid plexus epithelial cells (Bloch et al., 1985; Bloch et al., 1987). Experimental evidence indicates that Tf is essential for normal neural development, as Tf-knockout mice die shortly after birth unless supplemented with exogenous Tf (Dickinson & Connor, 1998; Trenor et al., 2000). Within the CNS, Tf plays a critical role in myelinogenesis, as demonstrated by impaired myelin formation in Tf-deficient mice and by increased myelination in transgenic animals overexpressing Tf (Espinosa de los Monteros et al., 1999; Saleh et al., 2003). Over the past three decades, work by our group has established apo-Transferrin (aTf) as a potent promyelinating and prodifferentiating factor. *In vivo*, intrathecal administration of aTf during a restricted postnatal period (PND 1–3) induces the expression of major myelin proteins, including myelin basic protein (MBP) and 2′,3′-Cyclic-nucleotide 3′-phosphodiesterase (CNPase), without affecting proteolipid protein (PLP) levels (Escobar Cabrera et al., 1994; Escobar Cabrera et al., 1997; Marta et al., 2000; Marta et al., 2003). This developmental window is tightly associated with the downregulation of the Tf receptor (TfR) as oligodendrocyte precursor cells (OPCs) mature, which suggests that cellular responsiveness to Tf is temporally constrained by receptor availability (Escobar Cabrera et al., 1994). Indeed, complete TfRdownregulation in mature OLGs renders them unresponsive to exogenous Tf, which reinforces the existence of a narrow period in which Tf exerts maximal biological activity.

Complementary *in vitro* studies show that aTf promotes OPC differentiation and enhances remyelination in experimental models of demyelination and hypomyelination (Adamo et al., 2006; Paez et al., 2002; Rosato-Siri et al., 2018; Silvestroff et al., 2012). More recently, the intranasal delivery of extracellular-vesicle-incorporated aTf has demonstrated robust promyelinating effects (Mattera et al., 2024) with translational potential. Mechanistically, Tf engages multiple intracellular signaling pathways associated with oligodendrogenesis. Our laboratory has shown that Tf activates Fyn kinase and downstream MEK/ERK and PI3K/Akt cascades, as well as the cAMP–CREB pathway (Pérez et al., 2013; Perez et al., 2009). The disruption of Tau-Fyn interaction impairs Tf-induced morphological differentiation, which confirms the central role of Fyn. Our findings support the idea that Tf functions as a multimodal developmental cue capable of coordinating cytoskeletal remodeling and transcriptional programs required for oligodendroglial maturation. Despite this progress, the fundamental mechanistic question remains of whether the promyelinating effects of Tf depend on its iron-transport capacity or else arise from intrinsic properties of the apoprotein. This issue has been difficult to resolve experimentally, as exogenously applied aTf can readily bind environmental iron before interacting with OPCs.

To overcome this limitation, we employed two structurally characterized, non-glycosylated human Tf mutants: (i) a quadruple mutant (TfY) lacking iron-binding capacity in both lobes, and (ii) a double mutant (TfK) that binds but cannot release iron. These tools allowed us to uncouple iron handling from the biological activity of the protein. Using the Oli-Neuoligodendroglial cell line, we demonstrate that both mutants promote morphological and molecular differentiation to a similar extent as wild-type Tf (TfWT). These results indicate that the prodifferentiating effects of Tf arise from the apoprotein itself and do not require iron transport or release. Therefore, Tf possesses iron-independent biological functions directly relevant to oligodendroglial maturation.

Altogether, our findings broaden the conceptual framework of Tf biology by demonstrating that its promyelinating action resides in the protein itself rather than its iron cargo. This evidence establishes Tf –and potential Tf-mimetic molecules– as promising candidates for the development of novel remyelination-enhancing therapies.

## Materials and Methods

### Materials

Biotin, sodium selenite, putrescine, progesterone, T3, Hoechst 33342, human aTf, and poly-L-lysine were obtained from Sigma-Aldrich (St. Louis, MO, USA). Human insulin (Insulatard) was purchased from Novo Nordisk (Buenos Aires, Argentina). Dulbecco’s modified Eagle’s medium (DMEM) powdered high glucose was obtained from Gibco (Waltham, MA, USA). Human Tf-Texas Red fluorescent conjugated (Tf-Txred) was purchased from Life Technologies (Buenos Aires, Argentina). The rest of the chemicals used were of the highest possible analytical grade.

### Oli-Neucells and Cultures

The Oli-Neu cell line (kind gift from Dr. PatriziaCasaccia) is an immortalized murine oligodendroglial cell line that recapitulates the morphology and physiological activity of oligodendroglial cells *in vivo* (Jung et al., 1995). Oli-Neu cells were grown in DMEM-high glucose supplemented with 10 ng/ml biotin, 30 nM sodium selenite, 5 µg/ml insulin, 100 µM putrescine, 20 nM progesterone, and 1% horse serum. Differentiation was induced only in the presence of 15 nM T3 in the culture medium. The cells were grown on poly-L-lysine-coated culture dishes and maintained at 37 °C in 5% CO_2_.

### DNA Encoding WT and Mutant Tf

DNA sequences encoding WT non-glycosylated humanTf and its mutants, TfY (Y95F/Y188F/Y426F/Y517F) and TfK (K206E/E534A), were obtained from Addgene (Watertown, MA, USA). The TfY mutant is unable to bind iron in either the N-lobe or C-lobe, while the TfK mutant is unable to release iron (Mason et al., 2004). The DNA, supplied as a dehydrated pNUT plasmid on filter paper, was rehydrated in 60 µl of a 10 mM Tris-HCl (pH 7.2) solution, and 2 µl of the plasmid solution was transformed into *E. coli* DH5αcells. Recombinant clones were selected using ampicillin, and plasmid DNA was subsequently isolated via small-scale preparation. The product obtained was analyzed by agarose gel electrophoresis with ethidium bromide. The migration patterns of the bands were consistent with the expected size of the pNUT-Tf plasmid, and the presence of the mutations was confirmed by DNA sequencing.

### Molecular Modeling

The structures of Tf were predicted with AlphaFold using the amino acid sequence of non-glycosylated human Tf and the corresponding TfY and TfK mutants, modeled both in the presence and absence of iron (Mason et al., 2004; Halbrooks et al., 2003; Jumper et al., 2021; Mason et al., 2002). The per-residue confidence metric (pLDDT) ranged from 70 to 90 for all models generated, which indicates high to very high confidence. The only exception was a short segment spanning residues E366-T369 in the inter-lobe linker, which showed lower confidence values (pLDDT 50–70), as expected for flexible regions. Structural images were rendered using UCSF ChimeraX (Pettersen et al., 2021).

### Transfection

Cells were plated at a density of 1.0 x 10^4^ cells/well on glass coverslips (diameter:1.2 cm) in multiwells a day before transfection. Transfections were performed using lipofectamine 2000 (Invitrogen, Waltham, MA, USA) according to the manufacturer’s protocol. Briefly, 2 µl of lipofectamine was used for every 0.8 µg of transfected DNA in 100 µl inserum-free culture medium. Transfections were performed using a total 0.8 µg of Tf plasmid WT, TfY, TfK or lipofectamine alone (control, no plasmid) for 6 hours, and transfection mixture was replaced with serum-containing medium. The plasmid contains a metallothionein promoter that regulates the transcription of Tf mutants in mammalian cell cultures.To induce expression of the wild-type and mutant transferrin constructs, cells were incubated with 50 µM ZnSO_4_ and 15 nM T3 for 48 hours. Because the objective of the study was to evaluate the biological effects of the expressed proteins, all subsequent immunocytochemical and functional analyses were performed after induction of transgene expression. Cell viability in response to 50 µM ZnSO_4_ was evaluated using the MTT assay, and no changes were observed at this concentration (data not shown). Additionally, the expression of the Tf mutants was evaluated by immunocytochemistry. For morphology analyses, cells were trypsinized and re-plated in coverslips with differentiation medium for 4 days.

### Immunocytochemistry

Once treatments were completed, the culture medium was removed from the wells, and cells were washed with PBS and fixed with 250 µl of 4% PFA for 20 minutes. The fixative was then removed and a wash with PBS was performed. The cells were blocked with 5% FBS in PBS-0.1% Triton- X100 (PBS-T) for 2 hours. The primary and secondary antibodies were prepared in 1% FBS in PBS-T and incubated overnight at 4 °C and for 2 hours at room temperature, respectively. After incubation with primary antibodies, cells were washed 3 times with PBS. For the observation of nuclei by fluorescence, Hoechst 33258 was added at a final concentration of 5 μg/ml in the secondary antibody dilution. Finally, the coverslips were mounted on glass slides with 15 µl of a mounting solution. The mounted coverslips were stored at 4 °C. The primary antibodies used were: chicken anti-MBP (1:500, Neuromics cat # CH22112), goat anti- mouse Tf (1:200, ICN), goat anti- human Tf (1:200, ICN), mouse anti- TfR1 (1:500 Thermofisher Cat #13-6800), rabbit anti-TfR2 (1:500, Bioss Cat # bs-9894R). The following secondary antibodies were used: Alexa Fluor 488-conjugated donkey anti-chicken IgY (1:1000, Jackson Cat #703-545-155), Cy3-conjugated goat anti-chicken IgG (1:1000, Jackson Cat #103-165-155), Alexa Fluor 488-conjugated donkey anti-goat IgG (1:1000, Jackson Cat # 705-545-147), Cy3-conjugated goat anti-mouse IgG (1:1000, Jackson Cat # 115-165-062), and Alexa Fluor 488-conjugated donkey anti-rabbit IgG (1:1000, Jackson Cat # 711–545152).

### Assay of Incorporation Human TfTxred

Transfected Oli-Neu cells were treated with Tf-Txred (100 µg/mL) for 5, 15, and 30 minutes at 37 °C to evaluate Tf incorporation. After treatment, cells were fixed and immunolabeled with MBP for immunocytochemical analysis.

### Microscopic Analysis

Fluorescence microscopy analysis was performed using multiple systems depending on the experimental design. Epifluorescence images were acquired using an Olympus IX83 microscope equipped with 40X and 60X oil immersion objectives, an Olympus BX50 microscope with 100X oil immersion objectives and spinning disc IX83 with 60X objective. Confocal imaging was obtained using a confocal Zeiss LSM880 microscope and an Olympus FV1000 with 60X oil immersion objective. Images acquired with the confocal microscope were digitally zoomed to enhance the visualization of selected areas. Epifluorescent images and confocal images were analyzed with Fiji software.

### Statistical Analysis

Three independent experiments were performed for statistical analysis using GraphPad Prism 8.00 software. Cells were imaged across 8 to 10 fields per condition. Data are presented as the mean ± SD. Two-tailed Student’s t-test and Kruskal-Wallis test followed by Dunn’s multiple comparison tests were used to determine statistical significance; ***p 0.001, **p 0.01, * p 0.05.

## Results

### Oli-Neu Cell Differentiation

**Figure 1.**
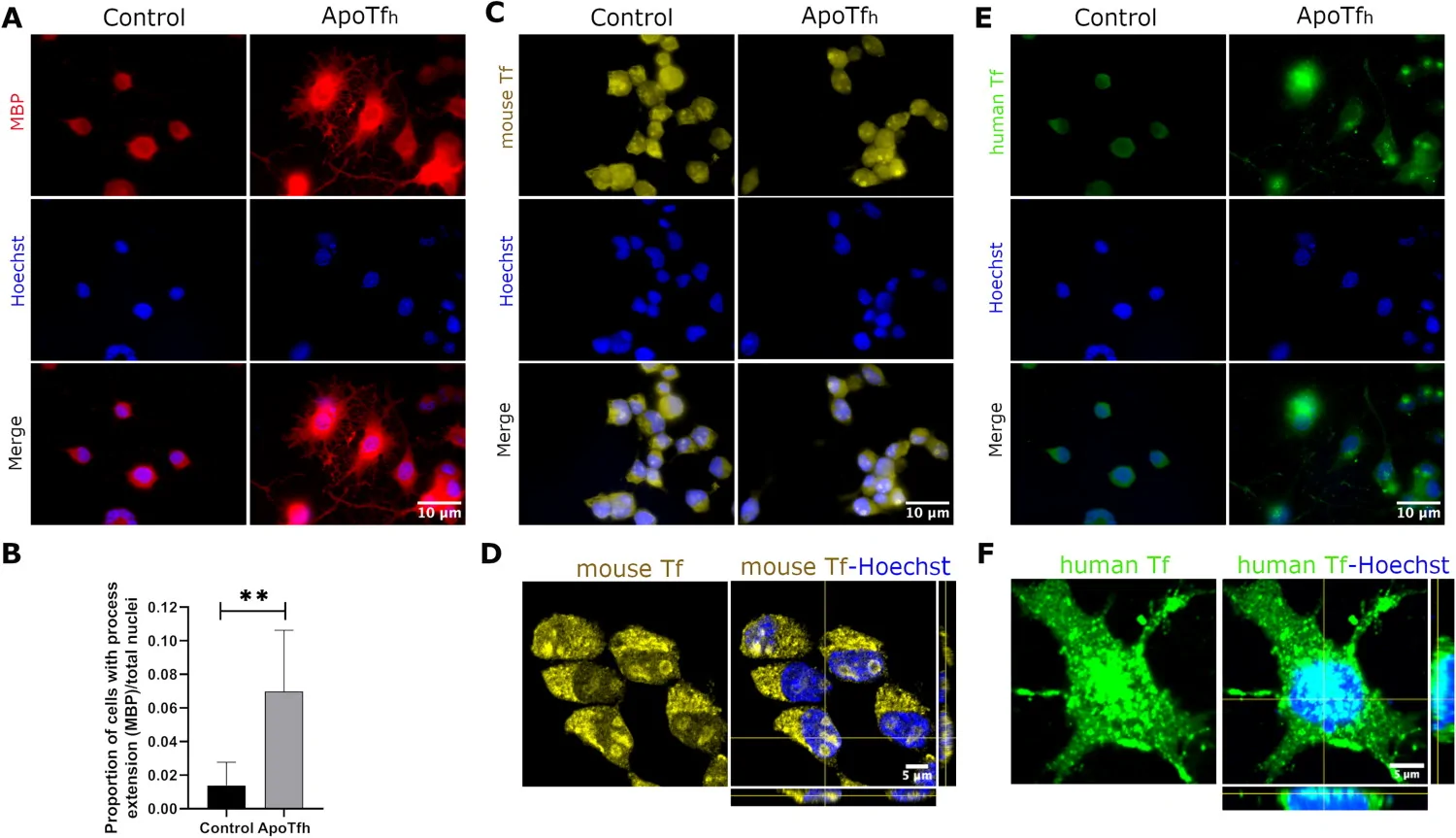
Humanapotransferrin-induced maturation of Oli-Neu cells.Oli-Neu cells were treated with 100 µg/ml human apotransferrin (ApoTfh) or left untreated (Control) for 4 days. Cells were immunostained for myelin basic protein (MBP) to assess oligodendrocyte maturation (A). Quantification of cells exhibiting process extension relative to the total number of nuclei is shown in (B). Cells were immunolabeled with an antibody against mouse transferrin, and the signal is shown using a yellow LUT (C), and confocal analysis revealed nuclear co-localization of mouse transferrin signal (D). Immunostaining with an antibody against human transferrin is shown in (E), with corresponding nuclear co-localization in (f). Specificity of the anti-human transferrin antibody was confirmed by Western blot, showing recognition of human but not murine transferrin (data not shown). Nuclei were counterstained with Hoechst.The yellow lines indicate the position of the orthogonal (XZ and YZ) projections generated from the confocal z-stack in Fiji/ImageJ.Thescalebar shown in themerged image applies to all images within the corresponding panel. The graphic represents the mean ± SD, **p < 0.01.

This study used cultures of an oligodendroglial cell line known as Oli-Neu which replicates OPC features and was established from O2A progenitor cells (Jung et al., 1995). We chose this cell line as an experimental model to investigate the differentiating effects ofaTf using both the wild type protein and two different mutated forms. Oli-Neu cells retained most of the phenotypical characteristics of primary OPCs even after several passages. Undifferentiated Oli-Neu cells grew in culture retaining their characteristic bipolar morphology (Figure 1A). Treatment with aTf and T3 for 4 days induced MBP expression and differentiation into OLG. The bar graph shows the changes induced in the number of MBP-positive OPCs with process extension (Figure 1B).

### Presence of Endogenous Tf

To determine whether Oli-Neu cells contain endogenous Tf, cultures were immunostained with anti-mouse Tf. Oli-Neu cells exhibited detectable levels of endogenous Tf, and the addition of human Tf did not alter endogenous Tf distribution (Figure 1C). Confocal reconstructions of z-stack images show that mouse Tf was present in the nucleus and cytoplasm (Figure 1D), while exogenous human Tf was distributed across the nucleus, cytoplasm, and processes (Figure 1E, F).

### TfR Expression in Oli-Neu Cells

**Figure 2.**
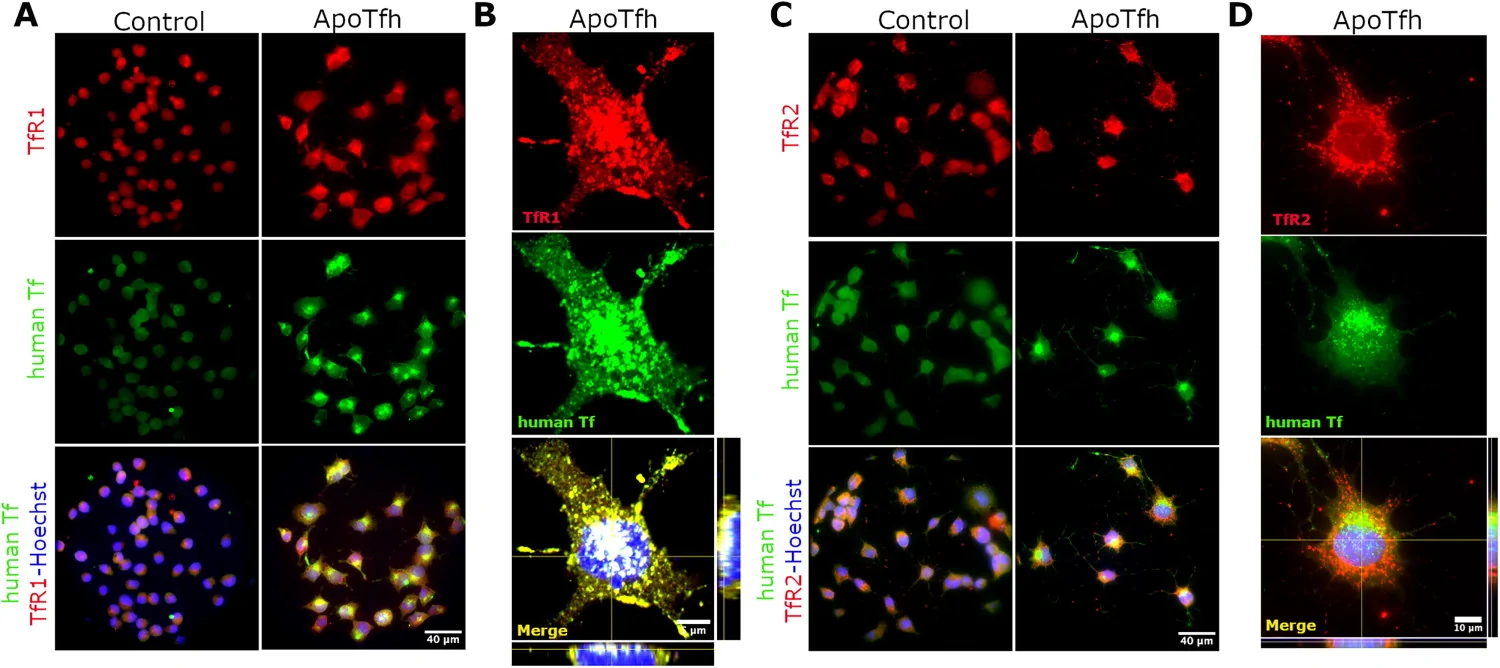
Expression of TFR1 and TFR2 in Oli-neu cells cultured with human apotransferrin (ApoTfh). Oli-neu cells were cultured for 4 days in the presence of or absence (Control) of 100 µg/mL ApoTfh. Cells were immunolabeled with antibodies against TFR1 (A, B) and TFR2 (C, D) (red), and with an antibody against human transferrin (green). Co-localization of TFR1 with human transferrin is shown in nuclei (B), whereas the distribution of TFR2 relative to human transferrin is shown in (D). The yellow lines indicate the position of the orthogonal (XZ and YZ) projections generated from the confocal z-stack in Fiji/ImageJ. The scale bar shown in the merged image applies to all images within the corresponding panel.

The presence of Tf receptor 1 (TfR1) was detected in Oli-Neu cells in the absence or presence of human Tf (Figure 2A). Confocal images show that human Tf and TfR1 colocalize in the nucleus and cytoplasm (Figure 2B). TfR2 is the second TfR-like molecule cloned by Kawabata et al. (1999) and localizes in lipid raft domains. While TfR1 is expressed in various tissues including the CNS, TfR2 expression is primarily confined to the liver (Fleming et al., 2000; Kawabata et al., 1999). However, in Oli-Neu cells, TfR2 was observed in a punctate pattern (Figure 2C, probably due to its localization in lipid rafts, but did not colocalize with human Tf (Figure 2D).

### Tf Expression in Oli-Neu Cells after Transfection

**Figure 3.**
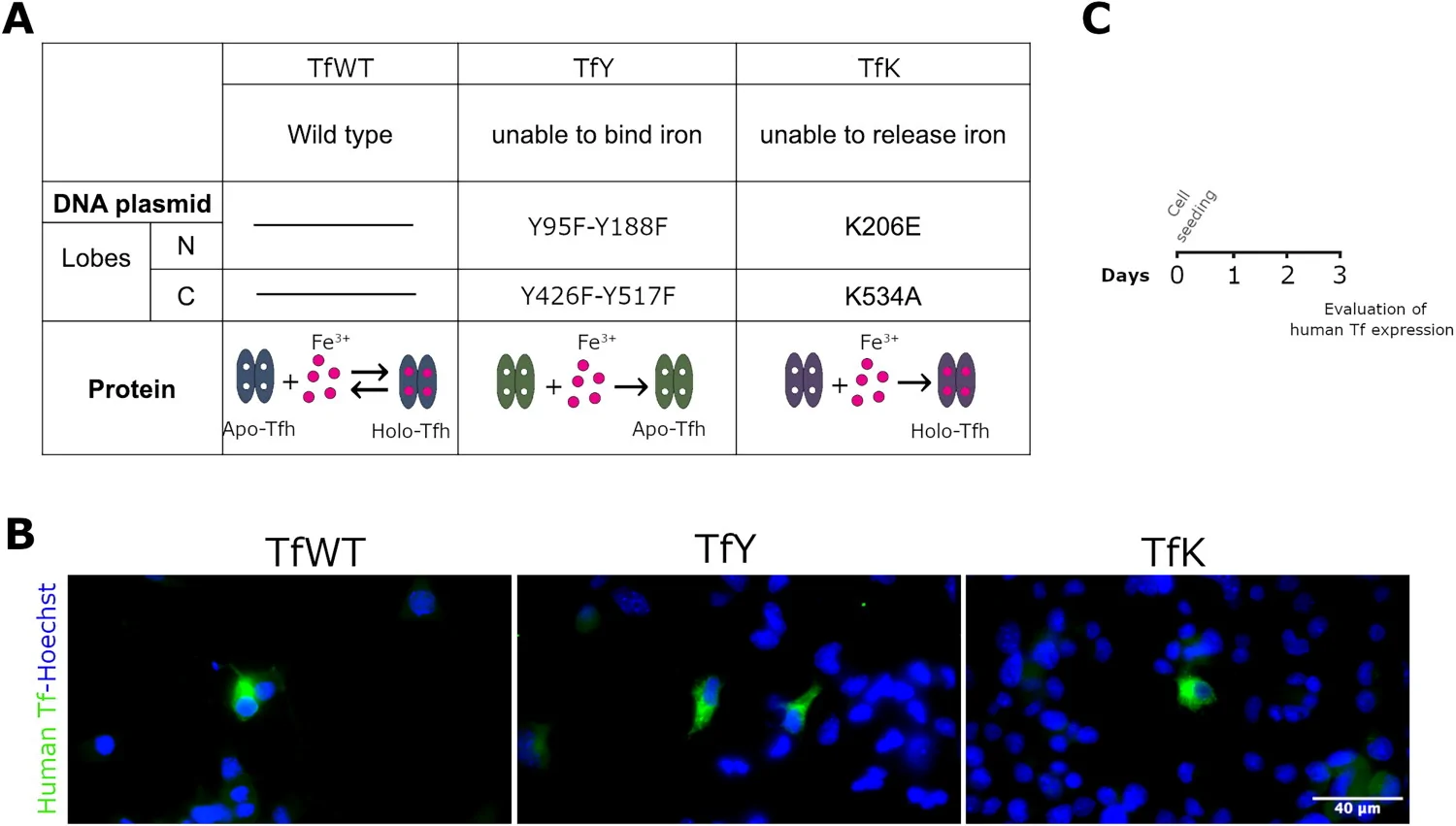
DNA plasmids encoding wild-type and mutant forms of human transferrin. DNA plasmids encoding wild-type human transferrin (TfWT), a mutant unable to bind iron (TfY), and a mutant unable to release iron (TfK) were used. The table summarizes the amino acid substitutions in the N- and C‑terminal lobes, along with a schematic representation of transferrin function in relation to iron binding (A). Expression of the mutated transferrin variants in Oli-neu cells was assessed 24 hours after induction. Oli-neu cells were transfected with plasmids encoding TfWT, TfY, or TfK, and the experimental timeline is shown in (B). Expression of the transferrin variants was induced with ZnSO_4_. After 24 hours, cells were fixed and immunolabeled with an antibody against human transferrin (green), and nuclei were stained with Hoechst (C). Representative images were acquired after ZnSO_4_ induction of transgene expression, corresponding to the experimental conditions used in all subsequent analyses.The scale bar shown in the merged image applies to all images within the corresponding panel.

Oli-neu cells were transfected with a DNA plasmid encoding WT human Tf, TfY, or TfK (Figure 3A). After 24 hours, cells were immunolabeled with anti-human Tf (green). All three Tf forms were expressed in Oli-Neu cells (Figure 3B). The representative images shown correspond to cultures following ZnSO_4_ induction of the metallothionein promoter, which was the experimental condition used throughout the remainder of the study.The experimental timeline used for transfection and subsequent analyses is depicted in Figure 3C.

### Different Transferrin Transfection into Oli-Neu Cells

**Figure 4.**
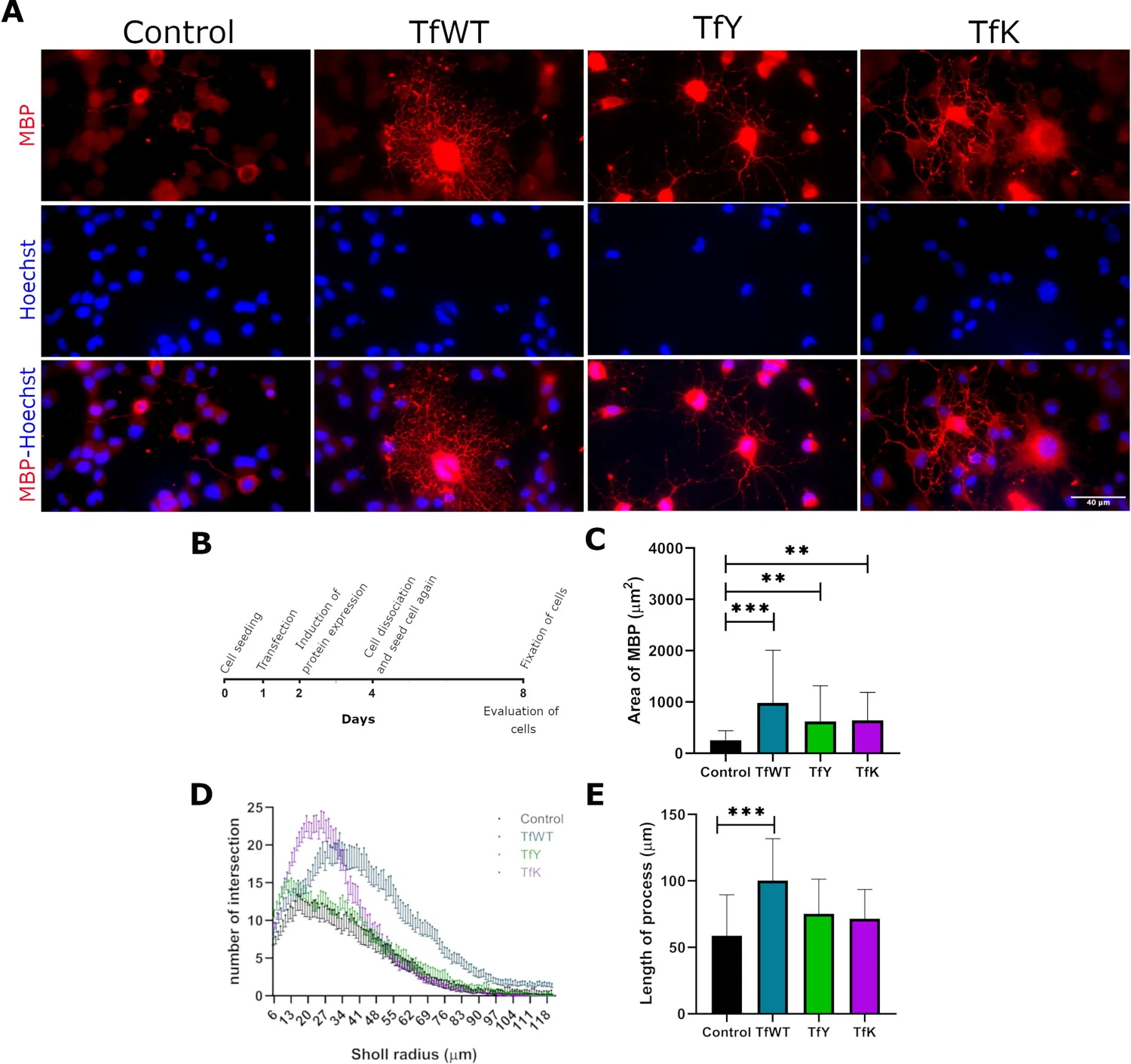
MBP expression in Oli-neu cells transfected with mutated and wild-type human transferrin. Oli-neu cells were transfected with DNA plasmids encoding wild-type human transferrin (TfWT), a mutant unable to bind iron (TfY), a mutant unable to release iron (TfK), or treated with lipofectamine alone (Control). After 48 hours of expression induction with ZnSO_4_, cells were dissociated using trypsin and re-seeded to monitor morphological changes. Cells were maintained in culture for an additional 4 days prior to fixation. Immunolabeling was performed using an anti-MBP antibody to detect differentiated cells, and nuclei were stained with Hoechst (A). Time scale used for experiments (B). Morphological changes were characterized through cell area quantification (C), Sholl analysis (D), and process length (E). The scale bar shown in the merged image applies to all images within the corresponding panel. The number of cells analyzed was 30 for each condition. The graphic cell area and process length represents the mean ± SD, ***p 0.001, **p 0.01, * p 0.05.

**Figure 5.**
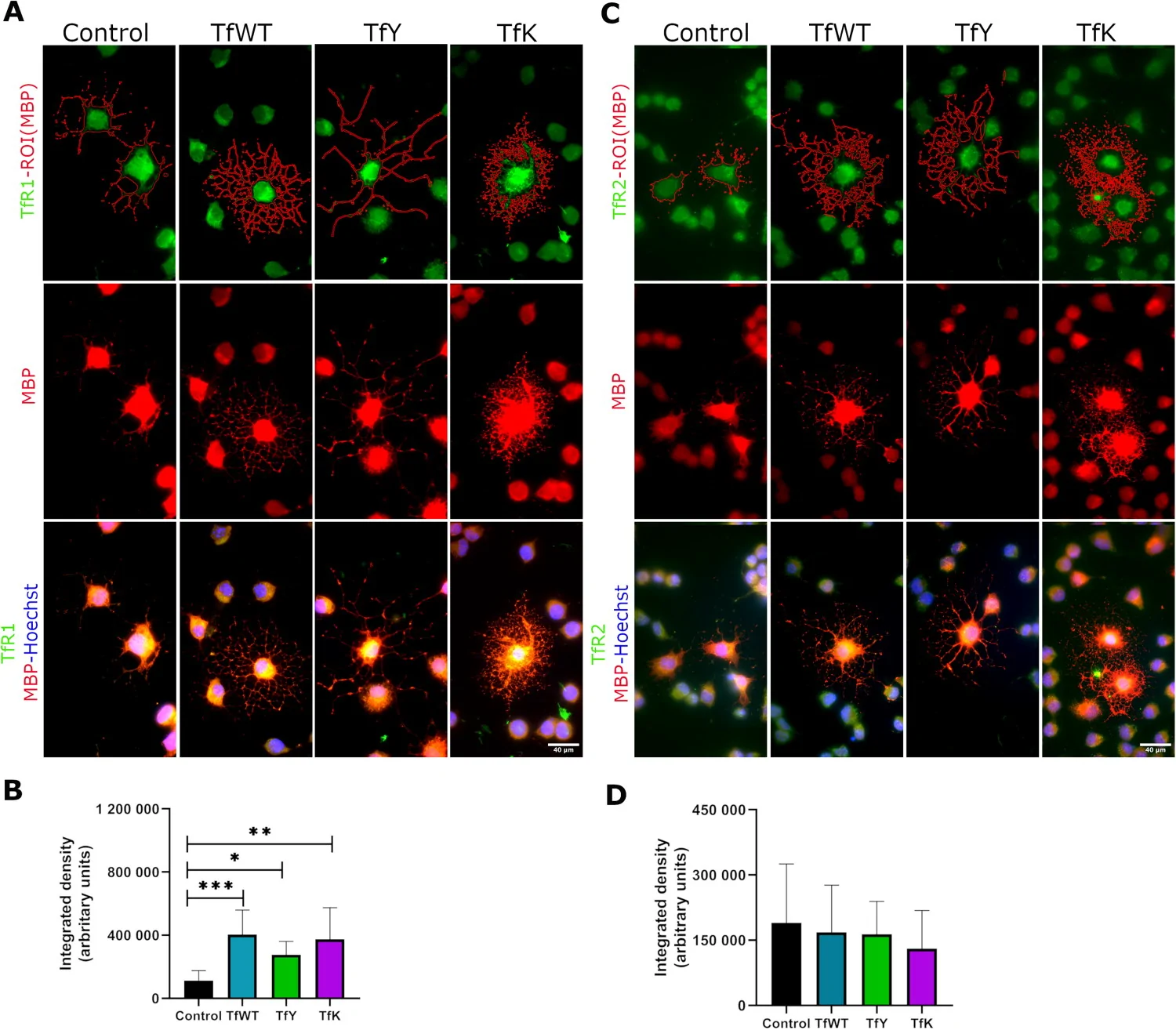
TfR1 and TfR2 expression in Oli-neu cells transfected with wild-type and mutant human transferrin. Oli-neu cells were transfected with DNA plasmids encoding wild-type human transferrin (TfWT), a mutant unable to bind iron (TfY), a mutant unable to release iron (TfK), or treated with lipofectamine alone (Control). After 48 hours of induction with ZnSO_4_, cells were dissociated with trypsin and re-seeded to monitor morphological changes. Cells were maintained in culture for an additional 4 days prior to fixation. Immunolabeling was performed using antibodies against TfR1 (A) and TfR2 (C) (green) to assess transferrin receptor expression, and anti-MBP (red) to evaluate oligodendrocyte differentiation. Nuclei were counterstained with Hoechst. TfR1 (B) and TfR2 (D) signal was quantified by measuring the integrated density within the region of interest (ROI), corresponding to the sum of the fluorescence intensity values of all pixels in the selected area.The scale bar shown in the merged image applies to all images within the corresponding panel.The number of cells analyzed was 10 for each condition. The graph shows the mean ± SD. ***p < 0.001, **p < 0.01, *p < 0.05.

The morphology of control Oli-Neu cells immunostained with anti-MBP is shown in Figure 4. Panels show the morphology of Oli-Neu cells transfected with human TfWT, TfY or TfK after 4 days of protein expression induction (Figure 4A,B). MBP area analyses showed differences between control cells and transfected cells with different mutated Tf constructs (Figure 4C). Figure 4D shows the results obtained in Sholl analysis in control and transfected cells. Quantitative analysis of process architecture revealed morphological differences between mutants. Notably, TfK cells exhibited higher branching density, as reflected by more Sholl intersections across multiple radial distances. In contrast, TfY cells displayed reduced morphological complexity with fewer intersections and a more compact arborization pattern as compared to TfK and control. Most interestingly, WT-Tf-transfected cells exhibited longer processes compared to non-transfected cells (Figure 4E). In addition, TfK and TfY cells showed changes in TfR1 labeling intensity (Figure 5A,B) but not in TfR2 (Figure 5C,D), which suggests a differential impact of the mutations on TfR1-associated pathways.Although these observations demonstrate reproducible differences in cellular morphology, the present experiments were not designed to determine whether they arise from intrinsic functional differences between the mutant proteins or from differences in protein expression, intracellular processing, stability, or transfection efficiency.

Despite a relatively low transfection efficiency (2-10%) under the different experimental conditions, transfected cells could be unambiguously identified in all three cases by the strong expression of the transfected construct.

Culture progression assays showed incomplete cell differentiation at 2 days in culture, as evidenced by the presence of O4-positive pre-OLGs. By day 6, cells displayed a more mature morphology consistent with advanced OLG differentiation (data not shown).

### Structural Integrity and opposite Conformational Locking of TfY and TfK

Although high-resolution structures of the quadruple TfY mutant are not available, the corresponding recombinant protein has been successfully expressed, purified, and biochemically characterized (Mason et al., 2004). Spectroscopic and electrophoretic analyses have shown that Y95F/Y188F/Y426F/Y517F lacks iron in both lobes yet behaves as a properly folded apoprotein, which is consistent with the preservation of the global Tf scaffold rather than destabilization or misfolding. Likewise, studies of mutations affecting the K206-containing triad demonstrate that impairing the iron-release mechanism does not disrupt the backbone architecture of the N-lobe, supporting the notion that iron-handling defects can occur without compromising global folding (Halbrooks et al., 2003).

**Figure 6.**
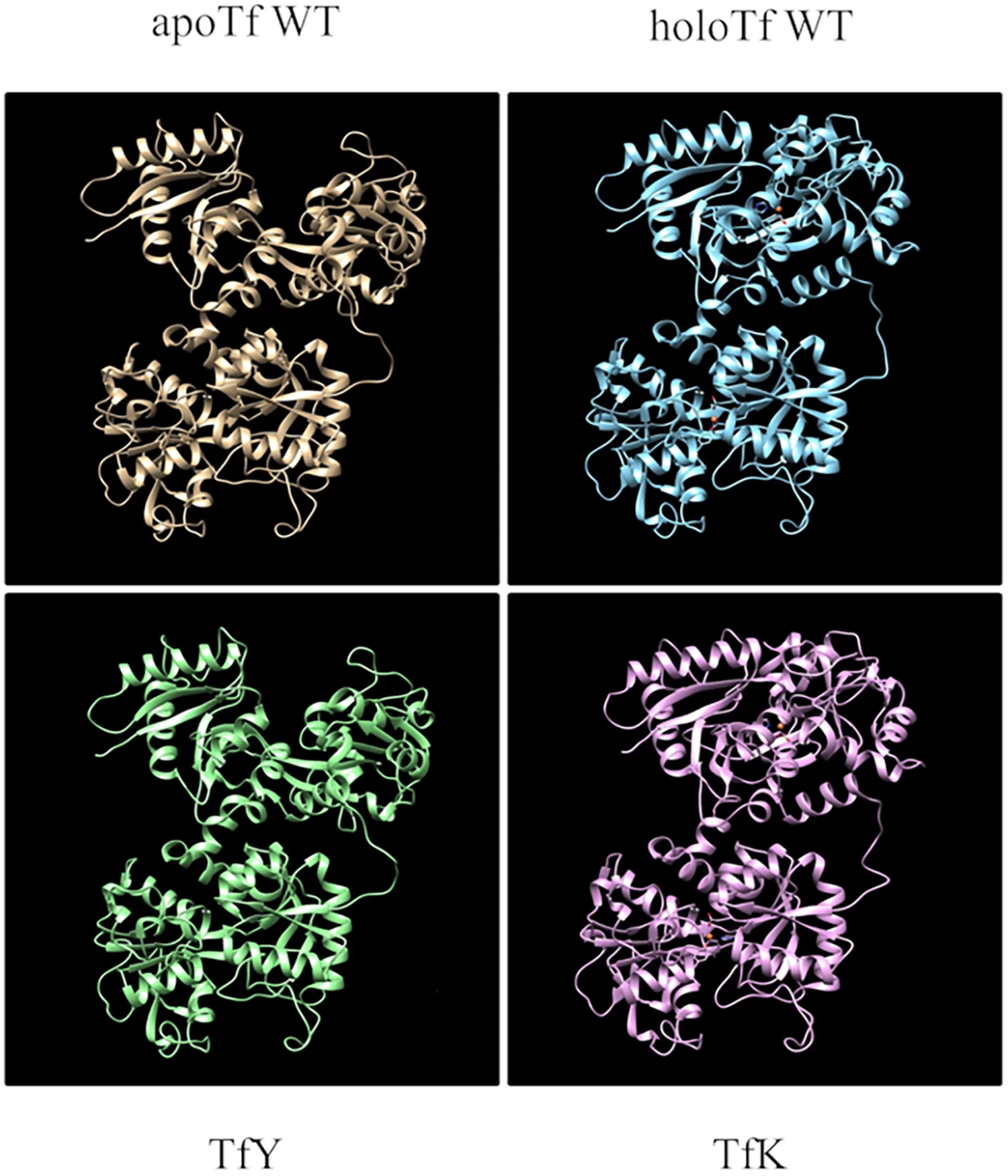
Structural models of wild-type and mutant transferrin variants. Ribbon representations of wild-type transferrin in the apo (apoTf WT) and holo (holoTf WT) states, and of the TfY (quadruple tyrosine-modified variant adopting an open conformation similar to apoTf), and TfK (a lysine-modified variant adopting a closed conformation similar to holoTf WT). The N-terminal (upper) and C-terminal (lower) lobes are shown in a consistent orientation across panels. Distinct colors are used to highlight each construct. All models display the characteristic bilobal architecture of transferrin, with no evidence of gross structural disruption in the mutant variants.

In agreement with these experimental data, our structural modeling revealed that TfY and TfK adopted opposite and mutually exclusive conformational states. TfY was locked in an open apo-like conformation in both lobes, which reflects its inability to coordinate metal. In contrast, TfK was locked in a closed holo-like conformation consistent with impaired iron release and the stabilization of the iron-dependent cleft closure. Both proteins, however, maintained the canonical bilobal architecture and inter-domain packing characteristic of native Tf (Figure 6).

These findings indicate that the mutations introduced selectively disrupt iron binding or release while preserving the global fold. Most importantly, our results also show that the resulting proteins are structurally trapped in opposite conformational states. The fact that both locked conformers retained the ability to promote OLG maturation supports the idea that this biological effect is mediated by conserved tertiary motifs that remain intact and functionally competent rather than by the dynamicapo-holo transition itself.

### TfTxred Incorporation

**Figure 7.**
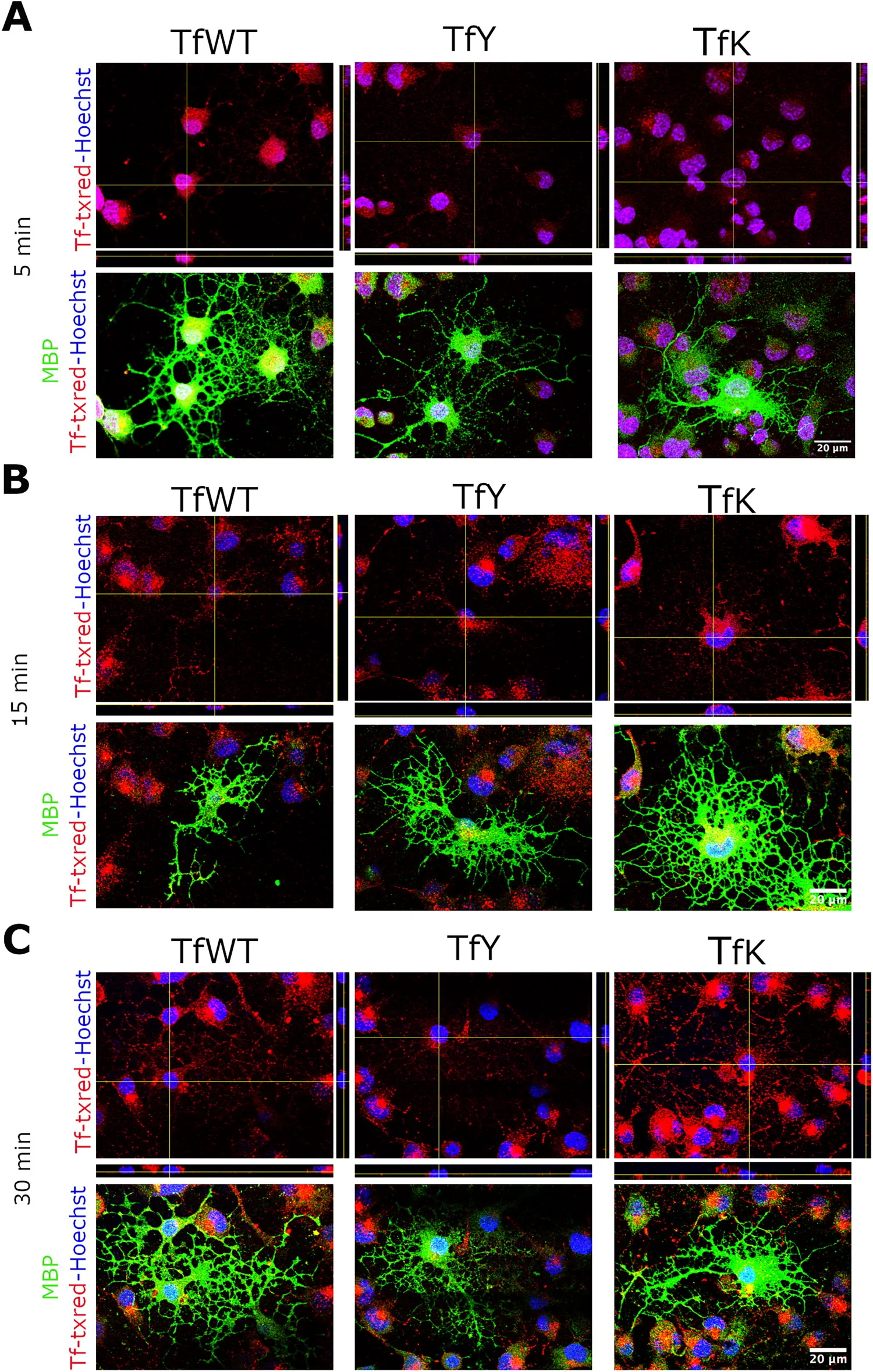
Incorporation of transferrin-Texas red in Oli-neu cells transfected with mutated and wild-type human transferrin. Oli-neu cells were transfected with DNA plasmids encoding wild-type human transferrin (TfWT), a mutant unable to bind iron (TfY), or a mutant unable to release iron (TfK). After 48 hours of expression induction with ZnSO_4_, cells were dissociated using trypsin and re-seeded to monitor morphological changes. Cells were maintained in culture for an additional 4 days.Prior to fixation, cells were treated with 100 𝜇*g*/*ml* of transferrin-Texas red for 5, 15, or 30 minutes. (A, B, and C, respectively). Immunolabeling was performed using an anti-MBP antibody to detect differentiated cells (green), and nuclei were stained with Hoechst (blue). The scale bar shown in the merged image applies to all images within the corresponding panel.

Oli-Neu cells in culture were transfected and analyzed 4 days after transfection and the induction of protein expression when cells were differentiated. TfTxred (100 µg/ml) was added to the culture medium, and uptake was examined at 5, 15 and 30 minutes (Figure 7A–C, respectively). TfTxred was observed in cells at the three time points studied, which indicates that transfected Oli-Neu cells retain normal functional activity.Interestingly, after 5 minutes Tf-Texas Red fluorescence was predominantly detected within the nucleus in both morphologically differentiated cells and cells that had not yet undergone morphological maturation (Figure 7A). By 15 minutes, the fluorescence had redistributed predominantly to the cytoplasm and cellular processes, with little nuclear signal remaining (Figure 7B). After 30 minutes, TfTxred was detected in the cytoplasm and cellular processes but not in the nucleus (Figure 7C).

## Discussion

Tf has traditionally been viewed as a systemic iron transporter essential for cell proliferation and mitochondrial activity. However, evolutionary data from invertebrates further support the concept that Tf-like proteins can acquire iron transport-independent roles. Several insect Tfs (e.g., Tsf3, Tsf4) lack canonical Fe³^+^-binding residues and instead appear to function in immunity, development, or cell communication (Geiser & Winzerling, 2012; Gorman, 2023; Najera et al., 2021). These data reinforce the notion that the Tf fold is highly adaptable and may mediate signaling functions unrelated to metal transport.

Multiple in vivo and in vitro studies by our group and others have shown that Tf also promotes OLG differentiation and myelination independently of iron delivery (Espinosa de los Monteros et al., 1999; Escobar Cabrera et al., 1994; Escobar Cabrera et al., 1997; Paez et al., 2002; Silvestroff et al., 2012; Mattera et al., 2024; Cheli et al., 2020). The present study extends this concept by dissecting the contribution of Tf’s structural properties from its iron-transport function. Using TfY, which cannot bind iron, and TfK, which cannot release it, we show that both mutants drive morphological and molecular maturation of Oli-Neu precursor cells comparably to WT-Tf. This finding suggests that Tf engages a promaturation pathway intrinsic to the apoprotein, functioning independently of iron metabolism and uncoupled from iron-dependent metabolic support.

A central implication of these findings is that two Tf molecules locked in opposite conformational states (open for TfY and closed for TfK) can both induce OLG maturation. TfY, lacking the tyrosine residues required for coordination, adopts an open, apo-like conformation in both lobes, whereas TfK, due to the disruption of the dilysine pair required for iron release, remains in a closed, holo-like state. Structural modeling validated prior evidence that, despite their opposing iron-handling properties, both mutants maintain the bilobal architecture and interdomain packing typical of native Tf. Although the AlphaFold models provide a useful structural framework for visualizing the predicted conformations of TfY and TfK, they should be interpreted within the limitations inherent to computational structural prediction. In the present study, the models were not intended to provide experimental validation of the mutant structures but rather to illustrate that the introduced mutations are predicted to preserve the overall bilobal architecture of transferrin. Importantly, these structural predictions are supported by previous biochemical studies demonstrating that both mutants retain proper folding while exhibiting selective defects in iron binding or iron release. Therefore, the AlphaFold analysis should be viewed as complementary to, rather than a substitute for, experimental structural characterization. Along the same line, biochemical analyses have shown selective loss of iron binding or release without evidence of global misfolding or instability (Mason et al., 2004; Halbrooks et al., 2003). Thus, the present findings suggest that the canonical apo–holo cycle is not required for the biological effect, and differentiation is triggered by structural determinants shared by both conformers. Mechanistically, these findings suggest thattertiary motifs or conservedsurface epitopes as the informational elements recognized by the oligodendroglial machinery, rather than the dynamic transitions associated with iron loading and unloading. These surfaces likely include regions at the lobe interfaces or receptor-contacting patches that remain intact in both mutated proteins.

These observations are consistent with a growing body of evidence indicating that several members of the Tf family have lost the capacity to bind iron in one or both lobes but retain distinct biological functions (Lambert et al., 2005). Indeed, the presence of Tf-related proteins with nonfunctional iron-binding sites underscores the need to redefine the functional landscape and evolutionary history of this protein family. Examples such as melanotransferrin, saxiphilin, and multiple insect Tfs demonstrate that the conserved Tf fold can support roles unrelated to iron transport. Our findings extend this paradigm by showing that human Tf itself can trigger OLG maturation independently of iron binding or release, which reveals a promaturation role embedded in structural features of the apoprotein.

The distinct cellular phenotypes elicited by TfY and TfK suggest that different transferrin conformations may influence oligodendroglial differentiation through iron-independent mechanisms. Although both mutants promoted differentiation despite opposite defects in iron handling, the molecular basis for these differences was not investigated in the present study. Sholl analysis showed that TfK induced greater branching complexity and more elaborate arbors, whereas TfY generated a more compact process architecture, despite both mutants increasing the MBP-positive area. These observations suggest that the two mutant proteins may not influence oligodendroglial maturation identically. However, the present study was not designed to determine the basis of these differences. In addition to potential differences in biological activity, alternative explanations, including differences in protein expression, intracellular processing, protein stability, or transfection efficiency, cannot be excluded. Consequently, these morphological differences should be interpreted cautiously. One possible interpretation of these observations is that the different conformational states adopted by TfY and TfK may differentially influence receptor behavior or intracellular signaling.Concepts such as conformational selectivity or biased agonism have been described for several receptor systems and could potentially explain the distinct morphological phenotypes observed here (Kenakin, 2019). However, because receptor trafficking, receptor kinetics, and downstream signaling pathways were not directly examined in this study, these possibilities should be considered. Our findings on TfR expression and localization provide a context for interpreting these ligand-specific effects. Our findings on TfR expression and localization provide a context for interpreting the biological effects observed in this study. Oli-Neu cells express TfR1, exhibit low levels of TfR2, and efficiently internalize exogenous transferrin. In addition, cells expressing TfWT, TfY, or TfK retained the ability to internalize transferrin, indicating that the canonical transferrin uptake machinery remains functional despite the mutations. These observations support the conclusion that the iron-independent promaturational effects of transferrin occur in the presence of an intact TfR1-mediated uptake system.

Although it is possible that the distinct conformational states of TfY and TfK differentially influence receptor trafficking or downstream signaling, these mechanisms were not directly investigated in the present study. Future studies combining receptor trafficking analyses, live-cell imaging, and biochemical signaling approaches will be required to determine whether these mechanisms contribute to the different morphological phenotypes induced by the two transferrin mutants.

Together with the preserved uptake of TfTxred in cells expressing the mutants, these findings suggest that the Tf cycle remains functional and that both mutants likely engage TfR1 in a similar qualitative manner. However, their distinct conformational constraints may modulate receptor trafficking dynamics, endosomal sorting, or nuclear translocation of Tf/TfR1 complexes. Although these pathways were not directly assessed, the fact that both mutants trigger maturation demonstrates that this promaturation pathway can be activated in the absence of iron, without precluding a modulatory role for iron under physiological conditions. These observations evoke conformational selectivity or biased agonism and point to the need for real-time signaling studies and proteomic profiling to identify the molecular partners differentially engaged by each mutant.

An additional observation of interest was the transient nuclear localization of endogenous transferrin, exogenous human transferrin, and transferrin-Texas Red shortly after uptake. Although transferrin is classically viewed as an extracellular iron transport protein that undergoes receptor-mediated endocytosis, transient nuclear localization has been reported for several receptor–ligand systems and may reflect receptor-associated intracellular trafficking or nuclear signaling events. In the present study, the rapid redistribution of transferrin from the nucleus to the cytoplasm suggests that nuclear localization is likely to be a transient process rather than a stable intracellular destination. At present, the biological significance of this observation remains unclear. It is conceivable that transient nuclear trafficking could contribute to signaling pathways or transcriptional regulation during oligodendroglial differentiation; however, the present experiments were not designed to investigate these mechanisms. Consequently, this finding should be considered an interesting observation that warrants further investigation rather than evidence of a defined biological function.

Our results also integrate well with previous work demonstrating that Tf directly induces MBP transcription without altering PLP mRNA, both in cultured OLG and in vivo. These studies showed that Tf increases MBP transcription specifically, whereas other prodifferentiation cues often act posttranscriptionally (Verity et al., 1990). Furthermore, intracranial injection of Tf has been shown to increase CNPase and MBP but not PLP (Escobar Cabrera et al., 1997). The present data, showing MBP induction by mutants incapable of iron handling, reinforce Tf’s role as a direct transcriptional regulator of myelin genes rather than a mere metabolic supporter of myelination.

Finally, our study shows that OPCs respond to a broad structural signature of Tf rather than to a specific conformational state. The fact that two mutants locked in opposite conformations both promote maturation challenges the notion that Tf primarily increases iron uptake by OPCs and instead indicates that OPCs decode conserved structural cues within Tf to drive cell-cycle exit, process extension, and membrane elaboration. Taken together, our findings support the conclusion that transferrin contains structural determinants capable of promoting oligodendroglial differentiation independently of iron binding or release. The precise molecular mechanisms through which these structural features are recognized by oligodendroglial cells remain to be determined and represent an important direction for future investigation.

A limitation of the present study is that all experiments were performed using the immortalized Oli-Neuoligodendroglial cell line. Although this model is widely used and reproduces many of the morphological and molecular characteristics of oligodendrocyte precursor cells, it does not fully recapitulate the complexity of primary OPC biology or the cellular interactions present in the intact central nervous system. Consequently, the present findings should be interpreted within the context of this experimental model. Future studies will be required to determine whether the iron-independent promaturational activity of the transferrin mutants is similarly observed in primary OPC cultures and in vivo models of developmental myelination and remyelination. Importantly, our findings are consistent with extensive previous work from our laboratory demonstrating that wild-type apo-transferrin promotes oligodendrocyte differentiation and remyelination in primary cultures and multiple in vivo experimental models, suggesting that the biological activity described here is likely to extend beyond the Oli-Neu system. The present study builds upon those observations by demonstrating, for the first time, that these promaturational effects do not require iron binding or iron release.

Another limitation of the present study is that oligodendroglial maturation was assessed using MBP expression together with morphological differentiation and process complexity. These are well-established markers of oligodendrocyte maturation but do not directly demonstrate the formation of functional myelin or effective axonal ensheathment. Therefore, our findings should be interpreted as evidence that transferrin promotes oligodendroglial differentiation independently of iron binding or release rather than as direct evidence of functional remyelination. Nevertheless, our results are consistent with previous studies from our laboratory demonstrating that wild-type apo-transferrin enhances myelin protein expression and promotes remyelination in both primary oligodendrocyte cultures and multiple in vivo models. Future studies using the mutant transferrin proteins in neuron–oligodendrocyte co-culture systems and experimental models of demyelination will be important to determine whether their iron-independent promaturational activity also translates into functional myelin formation.

Beyond their mechanistic significance, these findings may have important translational implications, as they position Tf as a bioactive, morphogen-like signaling molecule capable of modulating OLG fate independently of iron metabolism and demonstrate that its promyelinating activity can be uncoupled from iron transport. This notion opens the possibility of designing non–iron-binding Tf analogs or peptidomimetics that retain the structural determinants required for differentiation while avoiding disruption of systemic or local iron homeostasis. Structural comparisons among WT-Tf, TfY, and TfK offer an initial framework for identifying such motifs. Combined with targeted CNS delivery strategies, including intranasal administration, nanoparticles, or extracellular vesicles, these engineered Tf derivatives may provide new avenues for promoting remyelination in demyelinating diseases.

Funding This work was supported by a grant from the Agencia Nacional de Promoción de la Investigación el Desarrollo Tecnológico y la Innovación (PICT 2021-I-A-00747)

Author Contribution: JMP and JDC conception, design of the work, analysis and interpretation of the data and drafting and editing the manuscript. HPA Mutation of the protein. Design of the work and drafting and editing the manuscript. EChA Acquisition and analysis of the data Preparation of the figures. MG Acquisition of the data and PGF, MJP and AB acquisition of data. All the author reviewed and agreed with the data
